# The emergence of a new pilgrimage as an assurance game

**DOI:** 10.1098/rsos.250077

**Published:** 2025-09-10

**Authors:** Nicolas Restrepo Ochoa, Cristina Moya

**Affiliations:** ^1^ Anthropology, UC Davis, Davis, CA, USA

**Keywords:** game theory, assurance game, pilgrimage, collective rituals, institutional origins, opaque pay-offs

## Abstract

Pilgrimage has received long-standing scholarly attention, but most theoretical work focuses on how these rituals can be maintained, rather than the bigger puzzle of how they emerge. Using empirical inspiration from a new pilgrimage in Peru, we address this gap by outlining a theoretical framework more capable of accounting for how these phenomena get off the ground. We contend that the framework of an assurance game (a type of coordination game) captures the challenge of a collective ritual like pilgrimage emerging. By combining this assurance game with a model of Bayesian learning under uncertainty we illustrate how pilgrimage can be institutionalized on occasion. We further argue that our approach sheds light on the relationship between rituals and uncertainty, without having to make strong assumptions about individuals’ psychological needs.

## 1. Introduction

Current theories of pilgrimage [1,2] are good at explaining why these rituals endure once institutionalized, but they fall short in accounting for their emergence. We argue that the emergence of a new ritual like pilgrimage is better understood through the lens of an assurance game, a type of coordination game where a collective benefit is arrived at if, and only if, a certain level of collective action is reached. The key remaining question becomes how a society clears that initial threshold of enough actors choosing the social strategy, i.e. pilgrimage.

We begin by situating this analysis within the literature on the group- and individual-level benefits [1–4] of pilgrimage that help account for these collective practices. We then review some conditions that may facilitate pilgrimage emergence and the potential role of pilgrimage site entrepreneurs who have interests partly in conflict with those of pilgrims [1]. Then, we outline the ethnographic context that motivates our model: a recently emerged pilgrimage in the Peruvian Altiplano. We explain why the framework of an assurance game [5] is useful to illustrate the challenges of a pilgrimage emerging. We then extend this framework into a Bayesian simulation wherein individuals must learn about the pay-offs associated with going on pilgrimage and staying at home. To do so they combine their and others’ experiences with their prior beliefs. Inspired by our respondents’ motivations for pilgrimage, and echoing work on stochastic evolutionary games [6,7], we add a random frequency-independent pay-off that reflects variance in economic returns, but which people could interpret as a miracle granted due to their pilgrimage. The simulation allows us to explore how (i) prior beliefs about a ritual, (ii) the uncertainty of everyday activities, and (iii) social group size can affect the emergence and stabilization of a collective ritual like pilgrimage. To close, we add social conventions that constrain pilgrim decision-making to the model.

We find that collective rituals like pilgrimage can at times emerge if enough people test the practice, have lucky outcomes that year, and are therefore copied. This is more likely in environments where individuals engage in uncertain economic activities because unlikely, positive outcomes can more readily become associated with behaviours such as paying homage to a site, thus reinforcing the idea that a miracle has been granted. Similarly, weak priors can encourage early testing of the new practice and learning these positive associations. The structure of the assurance game capitalizes on this early, spurious relation by making it more likely that the pilgrimage practice is stabilized after the group crosses the critical threshold of pilgrims, after which the collective benefits of participation kick in. Without these frequency-dependent benefits, uncertainty could instead encourage the rejection of this newly established practice. We also find that it is easier for smaller groups to achieve the necessary proportion of pilgrims to get such benefits, and that some belief systems common in the Andean context facilitate the spread of new ritual sites.

## 2. Background

### 2.1. Motivations for going on pilgrimage

Social scientists have offered many theoretical accounts of pilgrimage [8]. The ubiquity and individual costs associated with these collective rituals have made it a prime target for scholarly attention. For example, why do pilgrims from around the world coordinate to go on the Hajj at the same time as each other, particularly given they will have to compete with others for limited space, pay higher prices, and sometimes face health risks due to sweltering temperatures? Some of the more influential theories, based on the work of Durkheim [9], appeal to this ritual’s capacity to engender a sense of *collective effervescence* that strengthens members’ sense of belongingness to the group [10]. In this tradition we find Turner & Turner’s [3] idea of *communitas*: the notion that pilgrimage acts as a liminal space where social hierarchies are broken, thus creating a togetherness among people who would otherwise not interact with one another. Empirical work has provided different lines of evidence supporting such claims, showing that during arduous collective rituals participants exhibit greater levels of synchronicity on markers like heart rate [11], that after such occasions they feel closer to the group [4], and that those who take part in these taxing behaviours act more prosocially [12].

In contemporary, evolutionary explanations of rituals like pilgrimage, the Durkheimian position is still dominant, though often tempered by a concern over the conflicts of interests between what is individually versus group-level beneficial. An underlying assumption in much of this work is still that pilgrimage does bring about certain collective benefits, and that these benefits pertain to a group’s ability to cooperate and endure [13–15]. However, greater emphasis is placed on how such arduous rituals might have additional individual-level functions like communicating one’s commitment to the group’s values. This line of argument is known as *the costly signalling approach to collective rituals* [15–17]. It notes that these practices are difficult, or not worth it, for non-believers, thus functioning as reliable cues for commitment to a set of beliefs [2,16]. A somewhat distinct signalling argument posits that it would only make sense for people with a longer-term stake in group membership to suffer these upfront pilgrimage costs. At the other extreme, people with no intention of staying in a particular moral community would receive no, or little, net benefit from pilgrimage. Therefore, the latter are less likely to engage in the arduous pilgrimage, making this activity a reliable signal of a person’s underlying commitment to being an active participant in a given community and being subject to their norm-enforcing institutions [18].

Other recent work has altogether challenged the idea of pilgrimage as a tool for promoting group identity. Pilgrimage, this line of work argues, is a site of contestation, where the interests of local elites and foreign worshipers clash, as do the interpretations of different groups of believers [19]. Not only do such approaches highlight the potential conflicts of interests between different actors, they undermine the plausibility of such rituals providing group-level benefits.

The above theories share an important shortcoming: they are not well-equipped to explain the emergence of collective rituals. The explanations that appeal to rituals’ capacities to engender *collective effervescence* or to serve as signals of commitment do not concern themselves with questions of emergence. Those that focus on pilgrimages’ contested meanings address an issue that would make starting a pilgrimage practice even harder. In one of the few papers that integrates these positions, Kantner & Vaughn [1] argue that there are two interlocking systems of signalling in the case of pilgrimage: religious leaders signal their causal grip on the world by displaying wealth, while a pilgrim’s journey is meant to exhibit an underlying quality of devotion, with a resulting system for maintaining group-level cooperation. Unfortunately, this argument still falls short of explaining the origin of the phenomena. All signalling theories of religion presuppose the existence of a connection between the behaviour—going to a shrine—and an underlying quality—e.g. commitment to a set of norms. This connection does not, by definition, exist at the emergence of a new pilgrimage. In other words, going by oneself on an arduous walk up a mountain is not a pilgrimage, and without communal agreement about its meaning it cannot serve as a signal of devotion.

Once established, a pilgrimage can indeed act as a cue for adherence to a group’s norms. Similarly, after a new spot has been identified as sacred, interested parties can undertake arduous journeys to the site to convince others of the sincerity [20], if not veracity, of their beliefs. However, at the moment of emergence of a new ritual, there is no agreement yet that the behaviour is a signal of commitment to the group. In this paper, we develop a model that illustrates the challenge of the emergence of a collective agreement, and a mechanism whereby it can nonetheless develop due to stochastic processes and social learning.

### 2.2. Exogenous circumstances giving rise to pilgrimage

Understanding the contexts in which pilgrimages arise can help refine our causal accounts of why they do so. Our model focuses on three factors that we believe relevant to the emergence of a collective ritual. The first is a population’s *prior belief* about the potential value of a new practice, especially when considered against the value of existing alternatives. In the case of the adoption of a new pilgrimage, people might surmise that staying home or going to an already established pilgrimage site has a better expected pay-off than trying something new. Alternatively, when people think that current practices are not working so well there may be relatively few opportunity costs to trying something new. In line with these expectations, research on millenarian movements suggests people are more open to new transformative ideas after cataclysmic events that render traditional practices less effective [21]. Genetically evolved intuitions that a practice is effective can similarly inform priors and bootstrap the spread of a religious practice [22].

The second factor we consider is the *uncertainty* in individuals’ everyday subsistence activities. There is ample work suggesting that rituals evolve as a way of managing uncertainty [23–25]. This suggests that at more uncertain times, more pilgrimage sites may emerge to satisfy this demand. However, such strong claims about human motivations merit more careful theorizing. While outcomes that are difficult to predict may motivate people to try out new practices, it is unclear why this uncertainty would not just as readily lead people to discard their value. Uncertainty may also disincentivize investment on the part of religious entrepreneurs who would offer new ritual opportunities [26]. Furthermore, many arguments about the benefits of religiosity or ritual conflate harsh conditions (i.e. ones that are predictably difficult) with uncertain ones (i.e. ones that are difficult to predict). Harsh conditions are often implicated in the literature on socio-economic gradients in religiosity within societies [27], and in historical accounts of conditions such as drought leading to a greater reliance on ritual [28]. However, it is a qualitatively different claim that unpredictable outcomes (e.g. fluctuations between drought and rainfall) lead people to ritual behaviours.

The last element we will consider is the *size of social groups*. At the longest historical timescales, reliable evidence of pilgrimage co-occurs with larger scales of social organization and regional integration [29,30]. However, these observations are challenged by the difficulty of detecting pilgrimage practices in the archaeological record [31], particularly those that do not preserve well because they do not result in monumental architecture (e.g. henges) or stable anthropogenic landscape features (e.g. Nazca paths). Indeed, the ethnographic literature suggests many lower-density foraging societies engage in rituals that emphasize the process of travelling to sacred sites [32]. In some of these accounts pilgrimage facilitates larger scales of social organization. In our model, we instead consider whether the scales at which people interact and learn from each other affect the emergence of collective rituals.

### 2.3. Interests of pilgrimage site actors

Not only do prospective pilgrims have to make strategic decisions about attending ritual sites, but they often do so in a landscape where religious authorities or locals at different sites have a vested interest in their attendance [1,19]. This means that a meaningful theory of pilgrimage not only must capture the reasons why pilgrims go on arduous journeys whose causal effects are at best opaque, it must also reflect that pilgrimage sites act as a locus of different actors who have a stake in the continued existence of the ritual. In our model, we will explore the implications of two kinds of rules that site-side actors might try to enforce, those about: (i) repeated attendance and (ii) information sharing.

Norms around attendance relate to a tension that pilgrimage sites must manage: the extent to which they entice many visitors versus maintain a stable stream of more dedicated adherents. While some associated practices may not entail a trade-off (e.g. both devoted and casual visitors may prefer a pilgrimage site with tasty food), there are some ways both goals may be incompatible. Most notably, having higher ritual expectations can act as a filter that reduces the popularity of a pilgrimage, but ensures that only those most devoted to the practice come and continue coming. There is a line of work that suggests that the spread and continued existence of strict religious groups, even against the backdrop of liberalization of religion, is explained precisely by those costs imposed on their members [18,33]. By imposing such costs, these groups can ensure a more active participation by all practitioners, thus increasing the overall benefits of membership [34], and even outlasting more lenient groups [35]. On the other hand, religious communities that have, often begrudgingly, accommodated diverse and syncretic practices, such as Catholicism [36], have been relatively effective at spreading, even if their followers have not been as doctrinaire. Being welcoming of casual practitioners versus restrictive to devoted ones may represent different pathways to success for a pilgrimage site.

A site trying to secure popularity might also develop strategies for reputation management. For example, a ritual may come wrapped up in a belief system about the steps necessary to ensure its effectiveness or about the information that can be shared. In experiments people are more likely to believe that repetitive, multi-step rituals are effective [37]. Demanding a pilgrimage ritual take such a complex form can make it harder to disconfirm its efficacy since a failure to have a miracle granted could be more readily attributed to a pilgrim having missed a step. Similarly, if the granting of miracles is based on the pilgrims’ own devotion, then having bad luck can be attributed to their insufficient faith. Not only would such belief systems be protected from disconfirming evidence, but they may also encourage asymmetries in the information being shared. Pilgrims may more readily share their successes and keep their failures private if the latter will be interpreted as evidence that they are either incompetent or lacking faith. In their analyses of the spread of inefficient medical treatments, de Barra *et al*. [38] contend that such biases in information transmission—only sharing successes—can lead to the widespread adoption of practices that are harmful or simply worse than their alternatives [38]. Hong & Henrich [22] make a similar argument regarding diviners under-reporting failures of divination. Thus, this asymmetric information transmission can help a collective ritual become established, even if it was perceived as ineffectual by most practitioners.

### 2.4. Ethnographic case study: Nuestro Señor de Pucara

The exogenous factors and site actors’ strategies, whose consequences we choose to model, are rooted in our work studying a newly emerged pilgrimage in the Peruvian Altiplano, Nuestro Señor de Pucara (NSdP). Around 2014 people started to congregate around a mountain ridge between two provincial towns to the north of Lake Titicaca. They were there to adore the face of Jesus Christ, now imprinted on the rock face. When and how the face got there is a matter of debate among locals. The most common origin myth, however, involves a miner who on his way to La Rinconada—a gold mine about a 2 h drive from the site—noticed the face and prayed to it. He would later return from the mine a rich man. Currently, thousands of people flock there in the first week of August to give offerings to Jesus’ apparition, and to the rock representations of other supernatural beings important in Andean traditions, such as the toad and eagle, that have also shown up at the site.

Our interest in examining a group’s prior belief in the efficacy of a potential new ritual is based on how NSdP fits within the local ecology of pilgrimage. Like other rituals in the Andes, NSdP can be considered a satellite ritual [39]: its principal dates are borrowed from a more established pilgrimage just across lake Titicaca, that of the Virgen de Copacabana. In fact, the similarities are such that many locals refer to this site as the ‘second Copacabana’. Many of its devotees have gone to both sites. In a regional sample collected in 2018, attending Copacabana is positively associated with having paid a visit to NSdP.^1^ The latter then emerges in a cultural context where people are perhaps predisposed to give a chance to a ritual with its features.

Our focus on the uncertainty of everyday activities stems from the idea that people may form illusory correlations between rituals and positive outcomes more readily in high-variance contexts. Residents near NSdP experience different degrees of economic variance depending on the extent to which they rely on mining, commerce or agropastoralism. Agropastoralists might go to a shrine and ask for good crops this season. Most of the time they will get what they expect—an average yet reliable yield—and thus they will not be surprised by the shrine’s intercession powers. Now, among miners, outcomes are much more variable. Again, in this context, most of the time the pilgrims will do just as well as they were doing before visiting the shrine. But in rare circumstances, they might suffer a tragedy or get a bountiful reward after visiting the site. If the latter happens then pilgrims might build an association between visiting the site and good fortunes at the mine.

Just as the origin myth highlights how NSdP rewards a miner with riches, most attendees seek supernatural help in achieving their goals. In the 2018 data, we see that individuals’ beliefs in the wish-granting efficacy of the site is an informative predictor of how many times they have visited.^2^ While this by no means indicates a causal relationship, it does suggest that wish-granting is of great importance to devotees. However, it is also plausible that repeated attendance brings about scepticism. Insofar as going on pilgrimage more times offers more opportunities for trial and error learning, each new visit provides new information about the site’s (in)efficacy. If pilgrims are judging the site’s wish-granting capacities, multiple visits followed by expected—or worse than expected—outcomes could potentially reduce their confidence in those capacities, while simultaneously mitigating concerns that one’s miracles were not granted for lack of piety.

The site-side strategies we explore in our model also find empirical grounds in NSdP. One recurrent theme in our interviews is that pilgrims must visit the site thrice for their wishes to be granted. While this expectation increases the burden of pilgrimage, such an attendance rule might ensure a steady flow of visitors. Second, pilgrims are less likely to share experiences when their wishes are not granted, compared with when they are. In a previous vignette experiment participants were asked whether pilgrims—whose miracles had or had not been granted—should tell others about their experiences (Moya *et al*., in preparation). The results reveal a bias towards positive information: those whose wishes were granted were more likely to be believed and encouraged to tell their stories. While this asymmetry is not directly norm enforced, failed requests for supernatural help are often interpreted as reflecting a lack of faith.

## 3. Methods

### 3.1. Pilgrimage as an *N*-person assurance game

We contend that the emergence of pilgrimage can be understood through the lens of an assurance game, usually known as a stag hunt game (SHG) [40]. In the traditional example, the players have two choices: hunting hare or hunting a stag. The first option can be done individually, and it provides a small but reliable pay-off h. Stag hunting, on the other hand, is a social strategy, and therefore requires coordination. In a two-player game, if both players play the stag strategy, they have enough people to take down a bigger animal and each get a pay-off s, where s>h. However, if only the focal agent decides to go stag hunting, they fail and receive no pay-off. In other words, the benefits of the social strategy can only be reaped if one can be assured that others will also engage in it.

**Figure 1.**
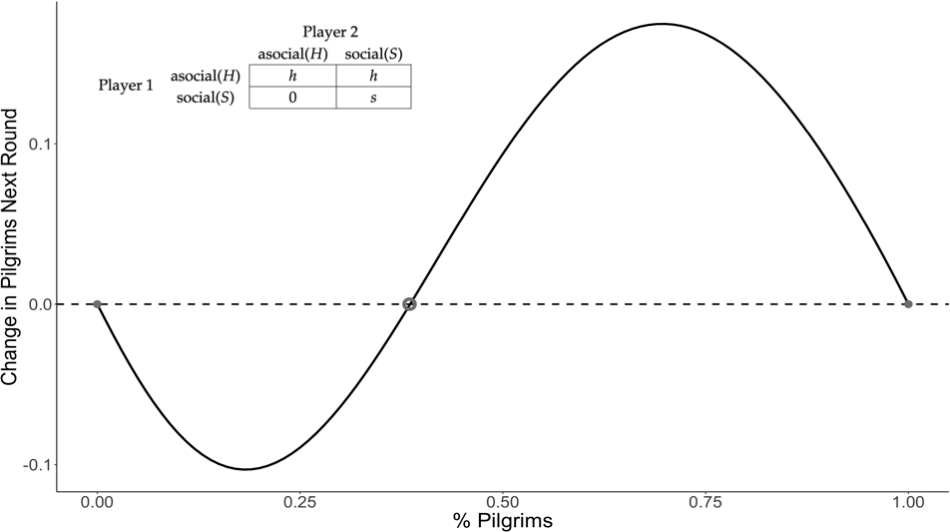
Simple replicator dynamics and pay-off matrix for two-person assurance game.

Figure 1 displays the replicator dynamics and the pay-off matrix for a two-person assurance game. The matrix contains the pay-offs from the perspective of Player 1. The 0 on the bottom-left cell represents the pay-off to the social strategy when coordination fails. The replicator dynamics—depicted by the solid line—in turn, capture how the strategies would spread across a population given a certain proportion of individuals playing the social strategy (pilgrims in our case). Imagine an infinite and well-mixed population containing a given proportion of agents playing the social strategy. From that population you sample two individuals randomly and see how well they do based on their strategy. More successful individuals reproduce and pass on their strategy. The *y*-axis captures the proportion of agents playing the social strategy after a round of pairings, given a proportion of agents already playing S. The two-person assurance game has two stable equilibria (represented by solid circles): either everyone plays the asocial or social strategy. It has one internal, unstable equilibrium (represented by the open circle), the proportion of social actors after which playing S becomes profitable and begins to spread across the population.

In the context of pilgrimage, h and s represent pay-offs to staying home and to going to the sanctuary if enough others pilgrims do so, respectively. The fact that h<s limits our interpretation of hare hunting to solitary activities that have lower pay-offs than a coordinated social activity. What then is the collective benefit that people draw from attending a pilgrimage that they would not get if they went to the same site alone? Pilgrimages can provide many tangible benefits that persist beyond the visit. These include opportunities to forge relationships with, or learn new information from, people from other communities. Though our interviewees seldom mentioned making new acquaintances at pilgrimage sites, this does not preclude it being a value of pilgrimage in other cultural contexts. At NSdP pilgrims often attend and engage in rituals at the site with family, friends or colleagues. While cementing such relationships can happen anywhere and at any time, doing so on the site’s feast day additionally offers the opportunities to share in nice food, live music, carnival games and the services of religious authorities such as shamans and a priest. Such services and attractions would not be provided without a critical mass of others and can help entice an acquaintance with whom one would want to strengthen bonds.

Pilgrimage may also provide less immediately tangible larger-scale group-level benefits that trickle down to the individual level. For example, the notion of *collective effervescence* [9] suggests that shared experiences renew people’s commitment to the group. More mechanistically, collective practices can make reputational networks or shared norms more apparent and engender trust, for example. For this initial model set-up, we need not commit to the substantive nature of the collective pay-off. What matters is that this pay-off is conditional on some coordination. Once we add Bayesian learning in the next section, we will need to restrict further what it means for pilgrimage to provide a collective benefit.

Our position resonates with the literature that conceptualizes religion more generally as a ‘club good’. This means that the benefit one draws from participation is conditional on the active participation of others and that said benefits—when achieved—are shared exclusively among members [18,33,34]. People who go by themselves to a site not yet recognized as a centre for pilgrimage will receive none of these communal benefits of the journey and have forgone the benefits of staying at home, h. Worst yet, they might be perceived as unreliable narrators or even as idolatrous.

Pilgrimage, of course, requires the gathering of more than two people. It is possible then to think about it as an *N*-person assurance game where the benefits of the joint activity are achieved only if a threshold number of people play the social strategy [5]. As we are interested in the emergence of pilgrimage, we start with the assumption that most people in the population do not go to the pilgrimage site. Rare innovators might venture on lone pilgrimages, but they represent the unlucky stag hunters who go out by themselves. Nonetheless, if enough people go to the site, all attendees will enjoy the collective benefits.

To understand the development of the *N*-person game, we envision again an infinite and well-mixed population with a proportion x of agents who are willing to play the social strategy (see for reference [5]). From that wider population, rather than pairs, now we sample groups consisting of *N* members. Let us say that for pilgrims to reap the collective benefits of their endeavour, there need to be at least M ritual goers in a given *N*-person group. Now, let k be the actual number of people who go to the pilgrimage. Thus, if k−M<0 then the threshold is not cleared, and the few enthusiasts do not reap the collective benefits of the ritual. Keeping the notation from above, we note that the function defining the fitness for those who do not go to the ritual is


$$\pi_{H}\left(k\right)=h.$$

This is an asocial strategy, so the pay-off is always the same, regardless of what other players do. For the ritual-goers, the pay-off is defined by


$$\pi_{S}\left(k\right)=\theta \left(k-M\right)s,$$

where $\theta \left(k-M\right)$ is a stepwise function that returns 1 if k−M≥0 or 0 otherwise. This means that the focal agent gets the benefit s if enough people show up and leaves empty-handed otherwise.

To calculate the average pay-off of each strategy, we have to take into account the different combinations of pilgrims and non-pilgrims we can get as we sample our *N*-person groups, and how likely those combinations are. We sum the pay-offs across the different compositions of size N of pilgrims and non-pilgrims, and we weight them by the likelihood of seeing those combinations given an existing proportion of pilgrims, x. Thus,


$$f_{H}=\sum_{k=0}^{N-1}\left(\begin{array}{c} N-1\\ k \end{array}\right)x^{k}{\left(1-x\right)}^{N-k-1}\pi_{H}\left(k\right),$$

$$f_{S}=\sum_{k=0}^{N-1}\left(\begin{array}{c} N-1\\ k \end{array}\right)x^{k}{\left(1-x\right)}^{N-k-1}\pi_{S}\left(k+1\right).$$

We are interested in how the proportion of pilgrims going to the site, x, develops across time. The replicator dynamics are determined by the variance of the trait and the difference between the expected pay-off of the two strategies,


$$x^{\prime }=x\left(1-x\right)\left(f_{S}-f_{H}\right),$$

where $f_{S}-f_{H}$ can be rewritten as


$$-h+\sum_{k=M-1}^{N-1}\left(\begin{array}{c} N-1\\ k \end{array}\right)x^{k}{\left(1-x\right)}^{N-k-1}s.$$

This expresses that pilgrimage will increase as a strategy if there is a positive difference between the pay-off of not attending and the benefits acquired by attending weighted by the probability of getting enough people to go to the site, conditional on the existing proportion of pilgrims.

The *N*-person assurance game provides a formal framework for approaching the emergence of a new collective ritual like pilgrimage and highlights the coordination that is required to get it off the ground. And yet it fails to achieve precisely what this article sets out to explain: how a new ritual can spread across a population of non-believers. The solid circle on the left-hand side of figure 1 states this plainly: the social strategy—going on pilgrimage—would not spread in a population where it does not already have some hold. For the social strategy to spread, there must be something that early adopters can gain, even when the collective benefit is not available due to lack of coordination. The framework of the assurance game is still useful, but it needs an additional element: a frequency-independent incentive that does not depend on coordination. We contend that in our case we can conceptualize this incentive as the promise of a miracle being granted. We build an agent-based model that adds to the framework of the assurance game a stochastic pay-off that, when positive, agents might perceive as a miracle. We show that the frequency-independent and frequency-dependent benefits can, in some cases, work symbiotically to stabilize new collective rituals.

### 3.2. Bayesian learning about miracles

In deciding whether to go on pilgrimage or not, prospective attendees not only assess the pay-offs captured by an assurance game, but also the probability that devotion at the shrine will grant them a wish. This is the *key element* we add to the framework of an assurance game: a purported miracle granting capacity of a site that adds to the possible perceived benefit of playing the social strategy, but that does not depend on how many others go. When a person—by chance—does well after going on a pilgrimage, they may consider themselves a recipient of a miracle, even if not enough others showed up to produce the collective benefits. The origin story of the lone miner who strikes gold reflects precisely this portion of the pay-off.

To model how individuals learn about the pay-offs associated with going to a new pilgrimage site, we turn to an agent-based simulation. The key addition to this model is that agents’ total pay-offs are affected both by the frequency-dependent (FD) pay-offs of the assurance game, and by the frequency-independent (FI) pay-offs of their everyday economic activities. The latter pay-offs are affected by environmental variance, which captures the uncertainty associated with people’s subsistence or economic activities.

This formalization can be regarded as an instantiation of the work on stochastic evolutionary games that examines the effect of random processes on the stability—or lack thereof—of certain strategies [6]. Just as small perturbations—like mutation—can push a system out of equilibria [6], environmental variance—in our case—may lead to the emergence of the collective strategy. In our model, the assurance game behaviour, pay-offs and learning happen in sequence such that—from the perspective of the pilgrim—there could be a causal link between the pilgrimage and particularly good—or bad—outcomes. This imagined causal link can motivate the focal agent, and those that learn from them, to adopt the social strategy, even before enough coordination exists. If this initial perturbation takes hold, the pilgrimage can emerge, even in a group where the asocial strategy was previously stable.

Here, we take the position that the shrine does not actually intercede to change the outcome of events, but that learners may misattribute variation in pay-offs due to everyday economic activities to the effect of miracles. Adding the possibility that people perceive miracles requires limiting the interpretation of the collective (FD) benefits of pilgrimage to those persistent benefits that can be conflated with the material (FI) pay-offs of one’s everyday economic activities. For example, a pilgrim might have a hard time discerning whether their business did particularly well this year because they made new social ties or learnt something new at the pilgrimage, because their colleagues cooperated more after this bonding experience, because they were motivated to work harder after publicly asserting their economic goals, or because God interceded on their behalf. On the other hand, we assume a pilgrim would be able to differentiate the FD pay-offs of finding food vendors at the pilgrimage site from the FI pay-offs of having more business later in the year. The conflation implied in the first set of examples is captured in our formalization by having agents add their assurance game and FI pay-offs. This reflects situations where collective pilgrimages have longer-term and causally opaque real-world benefits.

In the simulation, all agents start each time-step with two prior Gaussian distributions for the pay-off of each one of the strategies, staying at home and going to the site. At the beginning of each time-step, they take a random draw from their internal perceptions, and they play an assurance game using the strategy corresponding to the highest draw (see table 1). Agents then get pay-offs from playing the assurance game (FD), but they also get variable pay-offs from their everyday activities (FI). These pay-offs are summed, meaning agents conflate the FI benefits with those of the pilgrimage—e.g. potentially interpreting it as a miracle being granted or supernatural punishment. Agents then learn in a Bayesian fashion, updating both the mean and standard deviation of their priors about each strategy’s (pilgrimage versus staying at home) pay-offs. Then, they engage in social learning. They pick another agent at random and observe the strategy they played and the pay-off they received. Again they update their priors in a Bayesian fashion such that the information obtained through individual and social learning is weighed equally. Iterated over the time-steps, agents thus learn about the actual pay-offs for each strategy, becoming more certain about their priors.

**Table 1.** Model sequence.

step
initialization
1. the environment is initialized with an uncertainty given by $N∼(0,{PV}^{2})$
2. each agent starts with a prior for the two behaviours (social and asocial), defined by $N∼\left(\mu_{S},\sigma_{S}^{2}\right);N∼\left(\mu_{A},\sigma_{A}^{2}\right)$

modelling sequence
for each agent:
**1. choose behaviour based on beliefs:**
1.1 take random draw from one’s priors: $N∼\left(\mu_{S},\sigma_{S}^{2}\right);N∼\left(\mu_{A},\sigma_{A}^{2}\right)$
1.2 play the strategy associated with the distribution that produced the higher draw
**2. individually learn:**
2.1 frequency-dependent pay-offs: calculate pay-off based on whether enough others played the social strategy
2.2 frequency-independent pay-offs: add variance to payoff drawn from $N∼\left(0,{PV}^{2}\right)$
2.3 update priors for the chosen strategy based on $\text{pay-off}+N∼\left(0,{PV}^{2}\right)$
**3. socially learn:**
3.1 learn from a random demonstrator and observe their strategy
3.2 update prior for the strategy played by the demonstrator based on their pay-off

### 3.3. Model parameters

There are many parameters in this model—summarized in table 2—but we explore the effect of three key elements on the probability that pilgrimage takes off and is stabilized in the population. These variables of theoretical interest are (i) agents’ prior beliefs about the efficacy of the site (a function of the bottom six parameters in table 2), (ii) variance in the pay-offs of economic activities, *PV*, and (iii) the group size, *N*.

**Table 2.** Parameter list.

parameter	description
$T_{Max}$	number of turns
$R_{Max}$	number of rounds
N	number of agents
M	threshold for the social pay-off
PV	pay-off variance
AP	asocial pay-off
SP	social pay-off (when threshold cleared)
$\mu_{A}$	prior mean for the asocial strategy
$\mu_{S}$	prior mean for the social strategy
$\sigma_{A}$	prior variance for the asocial strategy
$\sigma_{S}$	prior variance for the social strategy
$\upsilon_{A}$	prior certainty for the asocial strategy (it can be interpreted as prior sample)
$\upsilon_{S}$	prior certainty for the social strategy (it can be interpreted as prior sample)

The first variable, agents’ priors, are shared across the population and always reflect an initial cultural expectation that staying at home is higher pay-off than going to a new sanctuary. However, these priors can be stronger or weaker at the start of the simulation, reflecting how certain individuals are about the relative costs of pilgrimage. The weaker the priors in the population the more likely some individuals are to try out the pilgrimage at its onset. Because we conceptualize these priors as cultural expectations rather than genetic ones, they only directly affect decisions on the first round. Therefore, we can convert this parameter to a more interpretable value; the number of pilgrims at the onset of the simulation. We systematically vary the standard deviation of the agents’ Gaussian priors to get three scenarios: an expected initial number of pilgrims of 1%, 5% and 10%.

The second element is related to the variance of the environment. This parameter is common to all agents, regardless of what strategy they play, and it is symmetric, meaning a more variable environment produces larger gains and losses. In other words, in a more uncertain environment, the social strategy can yield big pay-offs even if not enough people play it, and the asocial strategy can prove deleterious, even though the latter is on average higher pay-off. But variance goes both ways, and this means that it can shrink the collective benefit of the social strategy, even if enough people are playing it. We explore eight levels of variance ranging from 0.2 to 1.6, moving in increments of 0.2. Given the pay-offs of each strategy, this range covers a variety of scenarios, from ones where returns are quite certain to others where the strategies’ pay-offs are quite unpredictable.

The last element is the number of agents in the simulation, reflecting the scale of social interaction and of cultural transmission. The number of agents in a social group might play a big role given the stochastic FI pay-offs. In a small group, one unlikely outcome—branded as a miracle—might be enough to incite all agents into playing the collective strategy. However, for a large group, that one-time occurrence might not be enough to move the cemented opinions of most agents. We explore the simulation with 10, 50 and 100 agents. Every time, we let M—the threshold after which the social benefit is achieved— be $\frac{1}{3}$ of the group size. Treating this threshold as a proportion of a social group reflects the idea that many of the persistent collective benefits of pilgrimage probably depend on the relative, rather than absolute, number of people who act socially. For example, one would be able to forge bonds with most people in the relevant social group or find the small proportion of a community’s influential business owners if 40 out of 50 people went on the pilgrimage. Such connections would be more difficult to make if 40 out of 100 people went.

We then have 3×8×3=72 conditions. For each parameter combination, we run 50 rounds, each consisting of 300 time-steps and track the proportion of agents playing the social strategy.

### 3.4. Social conventions constraining decision-making

We finally modify the model to incorporate two rules reflecting site-beneficial belief systems that can constrain pilgrims’ strategies and learning. The first requires attending three times and the other allows only the spread of positive outcomes from the site (i.e. stifles reports of negative outcomes).

First, we implement a 3 year attendance rule wherein once a pilgrim has gone once, they commit to going twice more. This means that once an agent plays the social strategy, it will play the same strategy for the next two turns, without drawing from its internal distributions and comparing those draws.

Second, we consider the consequences of a normative system where only positive experiences are shared. We operationalize this in two ways; a (i) site-serving and a (ii) general positivity bias. In the former, among the agents who played the social strategy, only those whose pay-offs were better than the expected pay-off for staying at home could serve as potential demonstrators. In short, among the pilgrims, only the lucky ones would talk about their experiences. In the latter condition we consider that people in general are just more likely to report positive experiences. In terms of our model, this means that all agents—regardless of the strategy they chose to play—are taken out of the pool of demonstrators if their pay-off is below the expected pay-off of the asocial strategy.

## 4. Results

### 4.1. Pilgrim strategies

As noted above, the assurance game has two stable equilibria, and one internal threshold after which playing the social strategy becomes profitable [5]. Thus a first step to approach such models, is to think about how to make that threshold smaller—in other words, how to increase the basin of attraction of the social strategy. The easiest way to accomplish this is to increase the benefits of the social strategy and decrease the benefits of the asocial strategy. Note that, barring h<0, the social pilgrimage strategy still cannot invade a population of asocial actors, regardless of how much we change these pay-offs. In short, the basic assurance game model shows that pilgrimage can never emerge in a population with no pilgrims; the best the model can do is increase the basin of attraction of the collective strategy. For the collective pilgrimage strategy to invade we need to assume that agents perceive benefits to attending the site that are not FD (e.g. interpreting good business as miracles).

**Figure 2.**
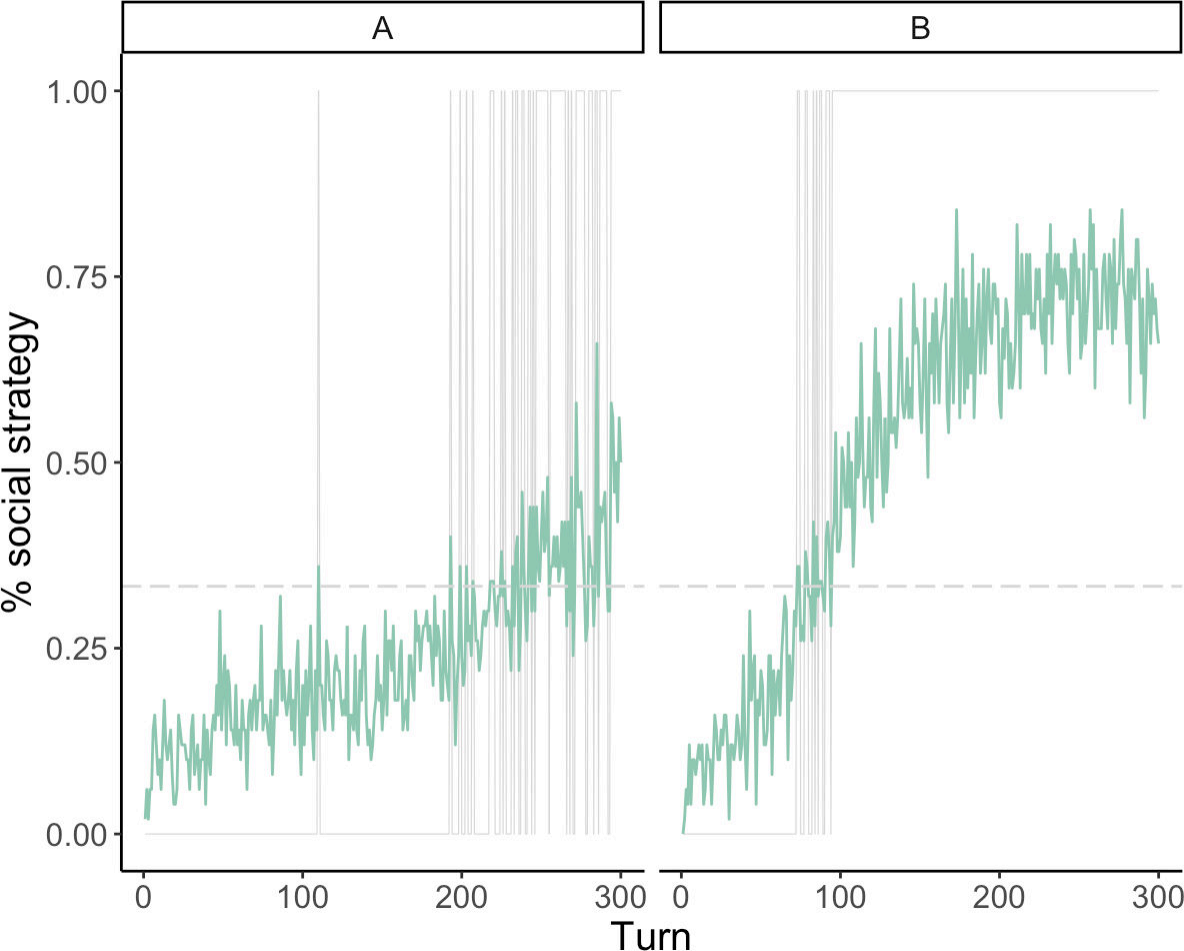
Two runs with different patterns of adoption. Both are taken from the parameter combination where the number of agents (*n*) is 50, the pay-off variance is relatively high (1.2), and the initial proportion of pilgrims is 10%. Grey, vertical lines represent the turns when the threshold *M* was cleared.

Adding this inherently stochastic process allows groups to *sometimes* cross the internal unstable equilibrium, enter the basin of attraction for the social equilibrium, and reach the stable equilibrium of only pilgrims. Figure 2 shows the trajectories of two runs that illustrate how this equilibrium can be reached. The *x*-axis captures the turns of the simulation and the *y*-axis the proportion of agents playing the social strategy. The green lines represent the proportion of agents who played the social strategy in each turn. The grey, vertical lines depict the turns where the threshold M was cleared—i.e. when the social benefit was available. We represent M with the dotted horizontal line. In figure 2A, we notice that the social strategy is rare until the end of the run, where it starts to increase slightly. As the simulation runs, the emergence of the collective ritual becomes increasingly unlikely because the agents—as Bayesian learners—start cementing their belief that the social strategy’s pay-off is approximately zero. It takes a lot of turns of successful coordination—notice the amount of vertical, dashed lines after approximately the 200th turn—towards the end of the simulation to start moving collective beliefs. Figure 2B, in turn, captures a scenario where the social strategy takes over the population very quickly. Here, we have an example of how a few early successes lead our Bayesian updaters to quickly change their belief and coalesce around the social strategy.

### 4.2. Exogenous circumstances

**Figure 3.**
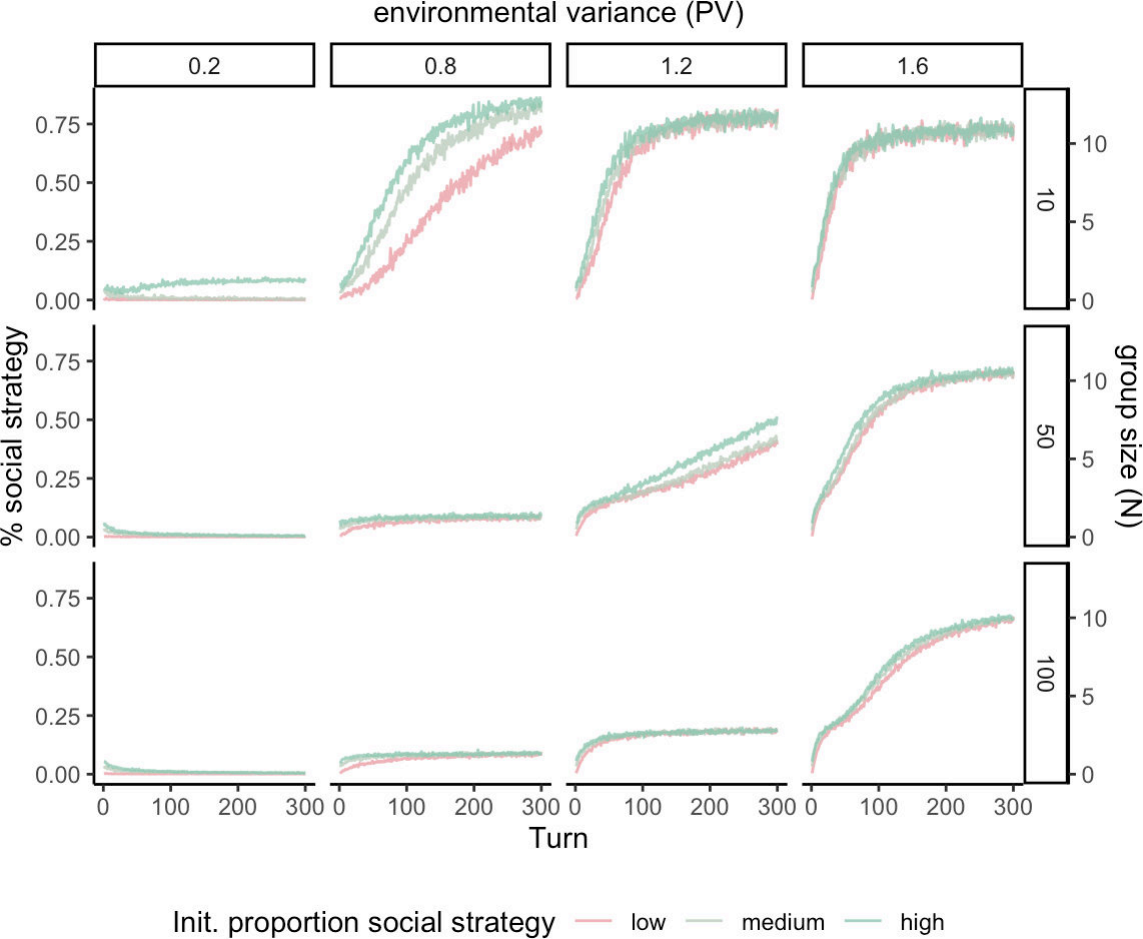
Proportion of agents playing the social strategy across parameter combinations. The columns represent different levels of environmental variance, the rows different group sizes, and the colours varying initial proportions of pilgrims.

Figure 3 captures how different exogenous circumstances affect the trajectories of adoption of the social strategy. As above, the *x*-axis and *y*-axis represent the turns of the simulation and the proportion of agents playing the social strategy respectively. The columns capture different levels of environmental uncertainty, and the rows different group sizes. The colours of each line represent the three initial proportions of agents that play the social strategy due to their having different priors.

Agents’ initial beliefs about the value of each strategy have some effects on the likelihood that pilgrimage emerges, and particularly how quickly it does so. We assume that agents begin with beliefs that staying at home is better than going on an untested pilgrimage, but that these beliefs vary in how strongly they are held. That means that the weaker the agents’ priors are that staying at home is more profitable than going on pilgrimage, the higher the initial frequency of pilgrims, and thus the more likely the social strategy is institutionalized (see differently coloured lines within each panel in figure 3). This is only a particularly notable effect in smaller populations with low environmental variance. These are the circumstances where weak enough beliefs at the beginning can make it easier to clear the threshold number of pilgrims necessary to make the social activity more profitable than the asocial one.

As hypothesized, environmental uncertainty, operationalized as increased variance in the FI pay-off, makes the emergence of the social strategy more likely and swamps the effect of initial beliefs (see increased average frequencies of social strategy from left to right panels in figure 3). Variance acts to increase the number of runs where a sufficient number of agents play the social strategy early on *and* get an unusually high pay-off from their economic activities. Under such circumstances their priors about the value of each strategy might shift. The same is true in turns when agents socially learn from unusually successful other pilgrims. In other words, variance facilitates the social strategy becoming associated with an overall high pay-off, even if there were no FD rewards to pilgrimage to start with. Nonetheless, there are diminishing returns to variance, at least in the smaller populations. This is because pay-off variance will encourage people to sometimes test the asocial strategy even after the FD benefits of pilgrimage kick in.

Overall, it is harder to get the social strategy institutionalized in larger groups. Larger groups require higher variances for the social strategy to go to fixation. This is because it takes a certain percentage of agents pursuing the social strategy to get the pay-off s. Imagine that 30% of agents are necessary to pass the threshold for getting the collective benefit in the assurance game. It is harder to coordinate 30 out of 100 people than it is to coordinate 3 out of 10 people. Specifically, the former is less likely because the mechanism responsible for jump-starting pilgrimage in this model relies on chance draws from prior beliefs about pay-offs and chance encounters with enough pilgrims.

### 4.3. Site-side adaptations

**Figure 4.**
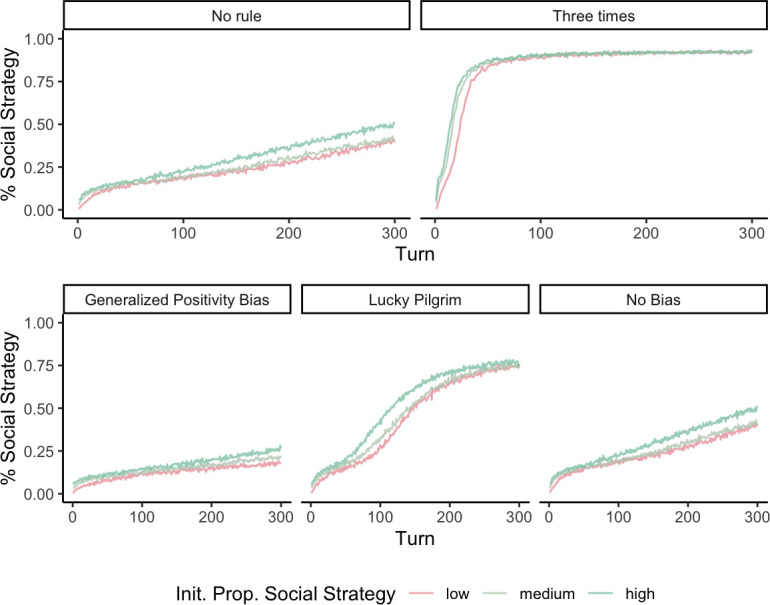
Trajectories of the social strategy for different attendance and transmission rules. The top panel compares regular attendance with the three-time attendance rule common in the Andes. The bottom panel compares different norms about what agents disclose depending on their luck after playing the strategies.

Figure 4 depicts trajectories of adoption of the social strategy under different attendance rules and social norms. The axes represent the spread of the social strategy as the simulation progresses, and the colours capture the initial proportion of agents playing that strategy. The columns, in turn, capture the different social norms. The top panel, for instance, compares the trajectories when different attendance rules exist. As expected, the three-time attendance rule speeds up dramatically the pace at which the social strategy goes to fixation.

The bottom panel, in turn, shows the effect of the different social transmission biases. Perhaps unsurprisingly, echoing De Barra *et al*.’s [38] findings, a site-serving ‘lucky pilgrim’ bias wherein the pilgrims only share their unexpectedly good pay-offs helps the spread of the social strategy. Incorporating this transmission bias makes it so that the social strategy reliably—and quickly—spreads across the population. However, a generalized positivity bias instead hampers the transmission of the social strategy. This is because, even though only the lucky pilgrims are sharing their stories, only the lucky ones among the agents playing the asocial strategy also serve as demonstrators. Early on in the runs, asocial role models are more common, meaning that agents can more quickly learn this positively biased information about the asocial strategy and thus cement their belief that pilgrimage is not worth it.

## 5. Discussion

It is estimated that over 150 million people go on pilgrimage every year, in practices that span all major world religions. This fact alone is puzzling given costs associated with rituals whose pay-offs are, at best, difficult to ascertain. However, understanding the origins of such practices is an even larger challenge than explaining their persistence.

We argue that current theories of pilgrimage fall short when it comes to explaining the emergence of this phenomenon. We use an assurance game to illustrate the difficulty of getting a new collective practice off the ground. Even if a society with pilgrimage does better than one without the practice, early adopters of pilgrimage will not receive the FD benefits that come from joining others at the same place, will suffer opportunity costs of travelling, and even risk being ostracized for unorthodox beliefs. In other words, individuals who adopt a practice like pilgrimage early on will suffer relative to those who stay at home, making it impossible for the practice to spread through selection or pay-off-biased learning.

Since the dynamics of an assurance game alone cannot explain the emergence of pilgrimage we incorporate another element: the wish-granting nature of these sites. We motivate our model with an ethnographic case study of a new religious pilgrimage in the Peruvian Altiplano, NSdP. Our participants in the surrounding region often respond that indeed NSdP must grant wishes given how many people are going. A common retort to our questions about the site’s efficacy is *‘one does not go just for fun’ (‘uno no va por gusto nomás’)*. There is, then, a perceived benefit that pilgrims derive that is not FD. As the model does not presume the site has any miracle-granting (or punitive) powers, agents’ perceptions of supernatural efficacy arise from Bayesian learning under conditions of uncertain pay-offs to everyday economic activities. When these are positive, agents may interpret them as the result of a miracle. People considering whether or not to go on pilgrimage may weigh their own previous experiences after going or not going to the site, and the experiences reported by others regarding their returns for that year. That is, agents update their beliefs both through individual and social learning. Since on average we assume the same FI pay-offs for pilgrims and non-pilgrims (i.e. there are no miracles), most runs result in an asocial (staying-at-home) equilibrium. However, there can be lucky runs where enough people have weak enough priors about the pay-offs of both behaviours that they try pilgrimage, are then lucky enough to do better than expected that year and by chance are picked up as possible models during the social learning phase. Importantly, while it is these lucky coincidences that can get the pilgrimage behaviour off the ground, it is the assurance game dynamics that stabilizes it into a tradition.

Having shown that merging an assurance game with Bayesian updating under uncertainty can sometimes lead to the emergence and stabilization of pilgrimage, we then explore the circumstances under which this is more likely. We vary three parameters; uncertainty in everyday economic activities, agents’ prior beliefs about the pay-offs of each activity, and the socially relevant group size. First, variance in the FI pay-offs increases the probability that the social strategy goes to fixation. Second, when agents have weaker prior beliefs about the pay-offs of pilgrimage and staying at home, more of them will go on pilgrimage early on, leading to a faster increase in the social strategy. Lastly, we notice that the effect of variance is moderated by the size of the group. As the groups grow, more variance is needed to get the social strategy off the ground. This is because we keep the threshold after which the collective benefit is acquired as a proportion, rather than an absolute number. For this reason, it is easier to overcome that threshold through chance events for smaller populations.

Lucky coincidences of pilgrims who do better in their everyday activities are more likely to happen in variable environments. The economic situation of pilgrims in the NSdP region may be such an uncertain context. In the Altiplano, people traditionally made a living from agropastoralism, with an increasingly mixed economy that incorporates commerce and mining. Because much of the commerce involves contraband, its pay-offs can be rather uncertain. However, gold mining is an even more variable source of income, and an increasingly popular one. La Rinconada, is a poorly regulated mining centre nearby where people may strike it rich, but may also succumb to debilitating, or even lethal, injuries. NSdP is found in a rural area where people still engage in subsistence agropastoralism, but it also lies on the recently asphalted road that connects the largest city in the region to La Rinconada, a 2 h bus ride away. This economic context means that the Peruvian Altiplano essentially has different populations facing different levels of risk. Our model can help explain why a pilgrimage site catering to those with more uncertain outcomes, like miners, would be more likely to emerge than one catering to those with more certain outcomes, like agropastoralists. Indeed, much ethnographic literature has documented the specific rituals miners adopt around the world [41,42], and some of our interviewees share the intuition that miners are particularly superstitious. However, our model also highlights that such uncertain environments also encourage some individuals to keep testing the asocial strategy each round.

The fact that larger groups are harder to coordinate is reflected in the lower probability that lucky runs stabilize pilgrimage when groups are larger. Whether this conclusion is warranted for the origin of pilgrimage depends on the ways we conceive of its FD pay-offs. The current instantiation requires a threshold percentage of a group needing to be on board to get the collective pay-off. This can represent the fact that finding the small percentage of useful social connections in a society depends on the percentage of people in a society who attend, rather than the absolute number who are there. Similarly, if there are social and reputational costs to going on a new pilgrimage, many collective benefits would arise only after a substantial proportion of one’s group was on board with the practice. However, if there were just a threshold *number* of people needed to get the collective benefits of pilgrimage, then larger populations would facilitate the development of pilgrimage. The people playing the asocial strategy would stay at home in such a situation and neither contribute to, nor detract from, the collective pay-offs. The fact that some of our interviewees are contemptuous of people who go to the site leads us to believe it is more appropriate to treat the threshold as a percentage of the population. That is, even if there were lots of people at a pilgrimage site, one would not want to join if a large proportion of one’s neighbours frowned upon it.

Finally, we modified the model to explore some cultural norms surrounding NSdP that might facilitate its institutionalization. We implement two biased transmission rules in our model reflecting a site-serving and a general positivity bias. The former is modelled after our participants’ tendency to particularly believe and encourage lucky, over unlucky, pilgrims’ stories. We operationalize it in the model as those agents who play the social strategy only sharing their pay-offs if they got lucky. This site-serving bias, unsurprisingly, helps the social strategy go to fixation much more quickly. By contrast, a general positivity bias where all agents regardless of strategy only share lucky outcomes, has a negative effect on the spread of pilgrimage by making agents learn positively biased information quicker about the asocial strategy. We have also found that a common rule for Andean sanctuaries is that one must visit them three times for one’s wishes to be granted. We build this attendance rule into our simulation and the results highlight that by having individuals attend at least three times, the site artificially bolsters the amount of coordination and this quickly propels the social strategy to fixation.

### 5.1. Broader applications

Though our focus is on the emergence of pilgrimage, we believe the model has broader relevance. This framework can shed light on the emergence of practices that are meant to have an effect in the world, whose efficacy is relatively opaque, and that bring about other long-term benefits when performed collectively. A collective gathering, like a musical concert, beneficial as it may be, lacks the element of trying to bring about a change in the world. Similarly, it is hard to imagine how a private ritual, like wearing an amulet before an important exam, can yield further benefits if others decide to join in.

A concrete example of a practice that would fall into this category would be a meditation retreat. At first, practitioners might be willing to go to a remote location to sit in silence, hoping for a calmer everyday after that. It may be that meditation is effective, but it also may be that, by pure chance, for some practitioners the time after the retreat is one of tranquillity. Those practitioner thus might form an association. If others notice that change, decide to follow suit and also happen to experience a shift afterward, then enough people might start showing up. After a group has been consolidated, other benefits can kick in, like forming connections with new teachers and being exposed to novel spiritual ideas. These collective benefits could be conflated with the presumed benefits of the practice itself, helping to sustain a recurrent tradition.

The model also provides further fodder for empirical tests. For example, when examining the possible relationship between uncertainty and adoption of new rituals, we may want to focus on measures of uncertainty that capture the difficulty of making causal attributions rather than ones that capture psychic needs. We can also examine how prospective pilgrims’ prior beliefs influence the likelihood that a community adopts a new practice. For example, it may not be surprising that evangelicals in the Altiplano are much less willing to grant that NSdP has supernatural powers given their strong priors against idols. Other predisposing or protective prior beliefs in the population are worth exploring. Finally, at a longer timescale, we hope the model helps provide conceptual clarity for anthropologists and archaeologists thinking about the role of population size in the development of collective rituals. If indeed larger populations are a predisposing factor for pilgrimage, our model would suggest it is despite the coordination costs associated with getting larger communities on the same page.

### 5.2. Limitations and future directions

Our model makes several simplifying assumptions for the sake of clarity and tractability, but which may nonetheless limit the generalizability of our conclusions and merit future investigation. First, we treat the FD and FI pay-offs in this model as fungible. In other words, agents can only see the *sum* of the pay-offs that pilgrims get from going to the site, and those they get from their everyday activities. We believe this is a plausible reflection of the ways people learn about the pay-offs of going on pilgrimage. Among religious rituals the fact that pilgrimage takes place away from one’s residential community makes it harder, particularly for social learners, to differentiate pay-offs due to everyday stochasticity and lingering effects of collective action. However, future models could examine whether the ability to discern the source of pay-offs erodes the spread of pilgrimage.

Second, one could imagine more complex relationships between population size, number of pilgrims and the collective pay-offs. For example, there could be even greater increasing returns to scale such that larger collective efforts could produce greater rewards, and perhaps even feedback on population dynamics [43]. Pilgrimages may both allow greater networking and bonding opportunities the larger they get and extend the relevant population size from which potential pilgrims are sampled. Alternatively, some of the rewards of pilgrimages might exhibit decreasing returns to scale after they get too large (e.g. accessing religious authorities or maintaining cultural cohesion becomes more difficult). This would add an element of anti-coordination to the assurance game dynamics and prevent a population from going to fixation. The fact that some global pilgrimages attract millions of attendees suggests at least some rewards see only increasing returns with number of social actors.

Third, we have discussed strategic considerations from the perspective of the site actors, but not modelled the coevolution of these rules with pilgrim strategies. For example, the site-benefiting rules of returning three times and only sharing positive information raises the question of why pilgrims would go along with these constraints. We could consider that individuals are aware of these upfront costs. Some of our interviewees have indeed internalized this attendance rule, but some also think strategically about the costs of the whole multi-year enterprise. Some even hesitate to commit to going precisely because of the costs of a 3 year commitment. In the language of our theoretical model, this means engaging in the collective strategy would entail forgoing three times the asocial pay-off—3h. This suggests a possible cost to the pilgrimage site of demanding too high an investment.^3^ Similarly, the fact that site-serving information is being disproportionately shared should increase prospective attendees’ epistemic vigilance [44]. A more complicated coevolutionary arms race between the pilgrimage site entrepreneurs and the prospective pilgrims may better capture the potential conflicts of interests between them.

Finally, our model does not take into account population structure. Previous work has examined stochastic coordination games when structure—in the form of relatedness—is included [45]. While relatedness helps the stabilization of the social strategy, local competition (inverse relatedness) leads to the evolution of polymorphic strategies [45]. Similarly spatial structure can facilitate or hinder the spread of social strategies depending on, for example, how social learning is implemented [40]. Other work has looked at the kind of networks that evolve when, under the framework of an assurance game, cooperators are more or less likely to acquire new connections compared with non-cooperators [46] or to select specific connections [40]. An underexplored avenue of work, however, is the kind of networks that would be most conducive for the emergence of the social strategy. For instance, in large, structured populations, we could imagine that the threshold of participation for gaining the collective benefit might depend not on the attendance of a proportion of the whole population, but rather of neighbours. Thus, small but dense substructures might more readily give rise to the collective ritual. Whether the ritual takes hold throughout the wider population might depend then on the connections between these substructures [47]. It is also plausible that the benefits of *communitas* [3] or *collective effervesence* [9] are more accurately captured as group-level outcomes that are copied from group to group. If this copying happens through a consensus building process or by a few authorities with influence over the population, this would entail fewer coordination costs for the early adopters. Understanding the kinds of structures that allow—or inhibit—the emergence of such collective rituals would be an illuminating next step.

## 6. Conclusion

We have argued that the assurance game provides a way of stabilizing those early chance associations that agents may learn between ritual participation and good fortunes, and thus help new collective practices go to fixation. Variance is a double-edged sword and extraordinarily good days at the mine are just as likely as hazardous ones. If it were just about variance, then, even lucky pilgrims would get quickly disenchanted after going to the site if their attendance were followed by less-than-ideal outcomes. However, the framework of the assurance game makes it so that if enough critical mass is garnered—through these early associations—then the benefits of the site do not hinge exclusively on its perceived causal powers but also on the very occasion of going to it. In other words, we move from a distribution centred at 0 to a distribution centred at s—the collective benefit. And this makes it likely for the collective ritual to become institutionalized.

Proposing a relationship between rituals and uncertainty is not new in social scientific theory. The key difference in our argument is that we are not appealing to any psychological need for a sense of mastery over the environment [48]. What we argue is more prosaic. More variable contexts are more likely to give rise to extraordinary outcomes which can, in turn, be associated with certain practices and/or rituals. If there is a way of capitalizing on those early associations—for example, because of the collective benefit provided in the assurance game—then those practices can spread and become institutionalized.

## Data Availability

All the necessary code and data to reproduce the analyses can be accessed through the following anonymous link: [49] The script ‘bayesian_stag_hunt.R’ is the agent-based model we use for the main analysis. The file named ‘parameter_exploration.R’ runs the model for the different parameter combinations and saves the result as ‘full_bayesian_parameter_exploration.rds’ in the ‘data’ folder. This is the file used to produce most of the figures on the ‘main_anon.Rmd’ file, which compiles the paper. The ‘supplementary_materials.Rmd’ file creates that document, using a csv file called ‘replication_df.csv’, which can be found in the folder. Supplementary materials are also available online [50].

## Footnotes

^1^
Full model specification and results included in the electronic supplementary materials.

^2^
Model specifications and results included in the electronic supplementary materials.

^3^
Elaboration included in the electronic supplementary materials.
